# A30 RECURRENCE RATES AFTER ENDOSCOPIC RESECTION OF LARGE COLORECTAL POLYPS: A SYSTEMATIC REVIEW AND META-ANALYSIS

**DOI:** 10.1093/jcag/gwab049.029

**Published:** 2022-02-21

**Authors:** R Djinbachian, C Rotermund, M Taghiakbari, M D Enderle, A Eickhoff, D von Renteln

**Affiliations:** 1 CRCHUM, Montreal, QC, Canada; 2 ERBE Elektromedizin GmbH, Tubingen, Baden-Württemberg, Germany; 3 Centre Hospitalier de l’Universite de Montreal, Longueuil, QC, Canada; 4 Klinikum Hanau, Academic Teaching Hospital of the Medical Faculty, Hanau, Germany; 5 CHUM, Montreal, QC, Canada

## Abstract

**Background:**

Complete polyp resection is the main goal of endoscopic removal of large colonic polyps. Resection techniques used for their removal have evolved in recent years and endoscopic submucosal dissection (ESD), endoscopic mucosal resection (EMR) with margin ablation, cold snare polypectomy (CSP), cold snare EMR and underwater EMR have been introduced. Yet, efficacy of these techniques with regard to local recurrence rates (LRR) compared to traditional hot snare polypectomy (HSP) and standard EMR remains unclear.

**Aims:**

We aimed to analyze LRR of large colonic polyps in a systematic review and meta-analysis.

**Methods:**

MEDLINE, EMBASE, EBM Reviews, and CINAHL databases were searched to identify prospective studies reporting LRR or incomplete resection rate (IRR) after colonic polypectomy of polyps >10 mm, published between January 2011 and July 2021. Primary outcome was LRR for polyps >10 mm.

**Results:**

6928 publications were identified, of which 34 prospective studies were included into the analysis. LRR for polyps >10 mm at follow-up (FU) up to 12 months was 11.0% (95% CI 7.1–14.8; 15 studies; 6259 polyps). ESD (1.7%; 95% CI 0–3.4; 3 studies, 221 polyps) and EMR with margin ablation (3.3%; 95% CI 2.2–4.5; 2 studies, 947 polyps) significantly reduced LRR compared to standard EMR without (15.2%; 95% CI 12.5–18.0%; 4 studies, 650 polyps) or with unsystematic margin ablation (16.5%; 95% CI 15.2–17.8; 6 studies, 3183 polyps).

**Conclusions:**

Local polyp recurrence after standard EMR of large colonic polyps is high. LRR is significantly lower after ESD or EMR with routine margin ablation, so that these techniques should be considered standard for endoscopic removal of large colorectal polyps. CSP, cold snare EMR and underwater EMR should only be used within clinical trials until more high-quality data regarding LRR becomes available.

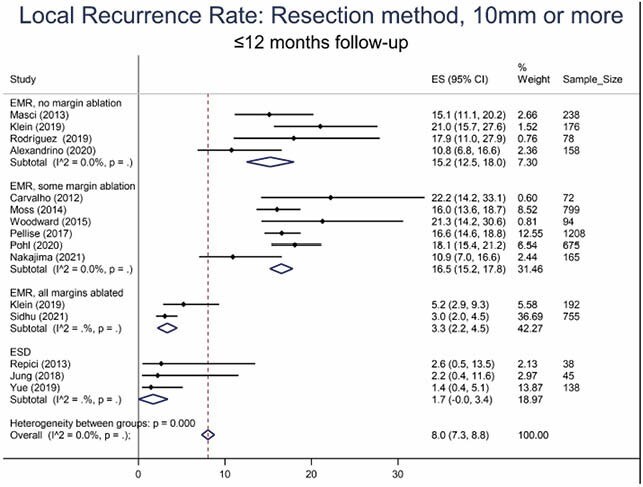

**Funding Agencies:**

None

